# *Youngiahangii* (Asteraceae, Crepidinae), a new species from Hubei, China

**DOI:** 10.3897/phytokeys.182.71063

**Published:** 2021-09-14

**Authors:** Qun Liu, Gui-Yun Huang*, Dai-Gui Zhang, Jian-Wen Zhang, Tao Deng, Zhi-Min Li1

**Affiliations:** 1 School of Life Sciences, Yunnan Normal University, Kunming 650092, Yunnan, China Yunnan Normal University Kunming China; 2 Rare Plants Research Institute of Yangtze River, Three Gorges Corporation, Yichang 443133, Hubei, China Rare Plants Research Institute of Yangtze River Yichang China; 3 College of Biology and Environmental Sciences, Jishou University, Jishou 416000, Hunan, China Jishou University Jishou China; 4 CAS Key Laboratory for Plant Diversity and Biogeography of East Asia, Kunming Institute of Botany, Chinese Academy of Sciences, Kunming 650201, Yunnan, China Kunming Institute of Botany, Chinese Academy of Sciences Kunming China

**Keywords:** Crepidinae, Hubei, molecular phylogeny, morphology, new species, *
Youngia
*

## Abstract

*Youngiahangii* T.Deng, D.G.Zhang, Qun Liu & Z.M.Li, **sp. nov.**, a new species of Asteraceae, is described and illustrated. It was collected in Wufeng County, Hubei Province, Eastern Central China. *Youngiahangii* is morphologically most similar to *Y.rubida*, but can be easily distinguished from the latter by capitula with 8–10 florets and the hairy leaf surface. Phylogenetic analyses, based on the internal transcribed spacers (ITS) and one chloroplast marker (*rps16*), showed that *Y.hangii* and *Y.rubida* were sister species with good support. The results of both phylogenetic analysis and the morphological data support the specific rank of *Y.hangii*.

## Introduction

*Youngia* Cass. (Cassini 1831) (Asteraceae; Cichorieae) is an East Asian genus with about 32 species (Babcock and Stebbins 1937; Shih 1997; Shih and Kilian 2011; Urbatsch et al. 2013; Deng et al. 2014; Peng et al. 2014, 2015; Ke and Chen 2016; Chen 2018). Most of the diversity of the genus is confined to China, and their diversity is especially high in the Hengduan Mountains (Peng et al. 2017). Most species of *Youngia* in China have a narrow distribution, especially several new species of *Youngia* which have been described in recent years (Deng et al. 2014; Peng et al. 2015; Ke and Chen 2016; Chen 2018). At the same time, there are two species of *Youngia* with incorrect taxonomic status. Youngiajaponica(L.)DC.subsp.longiflora Koh Nakam. & C.I. Peng is distinct from Y.japonicasubsp.japonica, which is supported by micromorphological and cytological evidence (Choi et al. 2020). *Youngianansiensis* Y.Z. Zhao & L. Ma was nested in the *Crepidiastrum* clade and, therefore, should be considered as *Crepidiastrumakagii* (Kitagawa) J.W. Zhang & N. Kilian (Shih and Kilian 2011; Peng et al. 2014).

A lack of conspicuous distinguishing morphological features makes the Cichorieae, especially the Crepidinae, taxonomically difficult (Babcock and Stebbins 1937; Shih 1997; Peng et al. 2013). The related genera, *Youngia* and *Crepidiastrum* Nakai (1920: 147) are particularly difficult to distinguish through morphological and palynological features like pollen morphology, the epidermis morphology of leaf and achenes (Babcock and Stebbins 1937; Shih 1993, 1997; Gao 2007; Sennikov and Illarionova 2008; Wang et al. 2009). First, the molecular phylogenetic studies supported *Youngia* to be part of subtribe Crepidinae Cass. ex Dumort. with the inclusion of subtribe Ixeridinae Sennikov (Kilian et al. 2009; Zhang et al. 2011; Tremetsberger et al. 2012). Then, the results of some investigations (Urbatsch et al. 2013; Liu et al. 2013; Peng et al. 2014) and the most comprehensive Crepidinae phylogeny (Wang et al. 2020) supported close relationships between *Youngia* and *Crepidiastrum* being sister groups to each other, but they are phylogenetically distant to *Ixeris*, *Ixeridium* and *Askellia*, and *Youngia* is polyphyletic. However, the circumscription of sections in *Youngia*, so far, still lacks molecular evidence (Babcock and Stebbins 1937; Shih 1997; Peng et al. 2014). Sennikov and Illarionova (2008) proposed to divide *Youngia* into four sections (*Youngia*, *Cineripappae* Sennikov, *Paleaceae* Sennikov [= *Mesomeris* Babcock & Stebbins, nom. inval.] and *Pinnatifidae* Sennikov), based on the sculpturing of the fruit surface, which was supported by the morphological structure of the achenes in the Cichorieae (Zhu et al. 2006; Zhang et al. 2013). These two studies currently lack the support of molecular results.

Due to the many floristic surveys dedicated to the flora of Hubei, a centre of Metasequoia Flora (Chen et al. 2018), many new species have been recently described from Hubei Province and the adjacent area (Lin et al. 2019; Zhang et al. 2019; Chen et al. 2020; Lv et al. 2020; Sun et al. 2020; Liu et al. 2021; Zhang et al. 2021). When conducting plant surveys in Houhe, we collected some interesting plants of *Youngia* from a cave in Wufeng County, Hubei (Fig. 4). After comparing them with the known species, we determined that they represent a new taxon, which we hereby describe as *Y.hangii*.

## Material and methods

### Morphological assessment

We compared the shape, lobes and size of the leaves, leaf surface, phyllaries, number of florets, achenes and pappus of the new collections with *Y.rosthornii*, *Y.rubida* and *Y.heterophylla* and with descriptions in literature, in the Herbarium of the Kunming Institute of Botany (KUN). Eight individuals of the new species were examined.

### DNA Sequencing and Molecular Analyses

For molecular analysis, we sampled a sample from one population of the unknown species and obtained 38 samples from 26 related species from GenBank (Appendix 1). Voucher information and GenBank accession numbers are presented in Appendix 1 Total genomic DNA was extracted from dried leaves using a Plant Genomic DNA Kit DP305 (Beijing, China) and used as the template in the polymerase chain reaction (PCR). Two sequences (ITS and *rps16*) were combined by Sequence Matrix v.1.7.8 for later analysis (Vaidya et al. 2011). Multiple-sequence was aligned using the programme CLUSTAL_W v.1.4 (Rédei 2008), followed by manual adjustment in BioEdit v.7.0.5.3 (Hall 1999). Gaps were treated as missing data.

Phylogenetic trees were constructed using Bayesian Inference (BI), Maximum Likelihood (ML) and Maximum Parsimony (MP). MP analyses were conducted using PAUP v.4.0a (Swofford 2004) by using a heuristic search, with random addition of 1000 replicates and tree bisection-reconnection (TBR). BI and ML analyses were conducted using MrBayes version 3.2 (Ronquist et al. 2012) and RAxML v.8.2.10 at the CIPRES Portal (https://www.phylo.org/portal2). The best-fit models of nucleotide substitution for individual data partitions were explored with Modeltest v.3.7 by Akaike Information Criterion (AIC). Using this procedure, GTR+I was identified as the optimal model. Bayesian tree topology was started from random trees and four Markov chain Monte Carlo (MCMC) simulations were run simultaneously. Runs were performed for 100 generations for a total of 10 million generations. The average standard deviation of split frequencies (< 0.01) was used to assess the convergence of the two runs. After the first ca.15% of trees were discarded as burn-in, the remaining trees were imported into PAUP* and a 50% majority-rule consensus tree was then produced to obtain posterior probabilities (PP) of the clades.

## Results

### Taxonomic treatment

#### Youngia hangii

Taxon classificationPlantaePhacopidaCheiruridae

T. Deng, D.G. Zhang, Qun Liu & Z.M. Li sp. nov.

51E31F18-D526-526C-84F8-B676EDD58ACD

urn:lsid:ipni.org:names:77219670-1

##### Type.

China. Hubei: Wufeng County, Renheping, 30°06'27"N, 110°16'31"E, karst cave of karst topography, 500–800 m alt., 5 August 2018, *Daigui Zhang & Qun Liu HAC 001* (holotype KUN (KUN1511675); isotypes KUN (KUN1511676), JSU (HHE 3256)).

##### Description.

Herbs, perennial, 20–35 cm tall. Taproot straight or slightly oblique, fleshy, with lateral roots (Fig. 2D). Stems erect, branched from the base, with sparse white simple hairs; stem base ribbed, with 1 or 2 leaves similar to basal leaves. Basal leaves crowded at the caudex base; petiole 2–3 cm long; blade oblanceolate, 6–18 × 2–4 cm, both surfaces short pubescent with white hairs 0.1–0.3 mm long (pubescence more evident on veins), bipinnate to pinnatifid, apical lobe halberd-shaped, apex acute-acuminate, margin deeply lobed; lateral lobes 5–10 pairs, opposite or slightly skewed, irregularly halberd-shaped (lateral lobes tapering to the base, serrate, lowermost lobes narrowly triangular), often with 1–3 pairs of triangular or oblique-ovate lobes between lateral lobes. Synflorescence corymbiform; capitula 7–10. Involucre ampullate, 3–4 mm long, 3 mm in diameter. Phyllaries in 4 rows, greyish-green; outer phyllaries 5–7, ovoid-triangular, ca. 1 × 1 mm, apex acute; inner phyllaries 7–9, lanceolate, 2–4 × 0.5–1 mm, margin white-membranous, apex acute; florets 8–10, ligules 4–6 × 1–2 mm, teeth 0.2–0.4 mm long, tube 3–4 mm; anther tube ca. 2.5 mm long; style branches ca. 0.5 mm long. Outer achenes black, fusiform, 2 mm long, apex attenuate to shortly beaked and expanded again into the pappus disc; ribs 12–14; pappus white, bristles rough, 3 mm long; inner achenes similar to the outer ones, 2.5 mm long.

**Figure 1.**
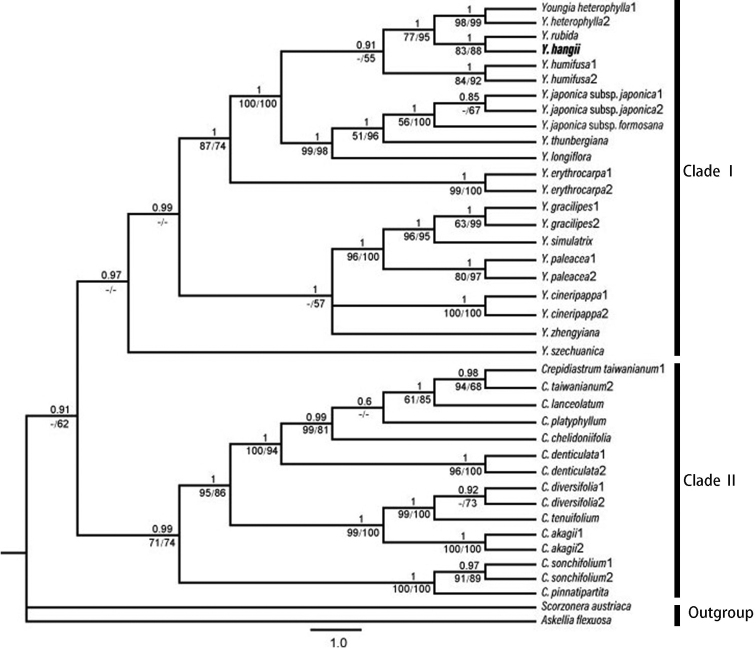
Bayesian consensus tree of *Youngiahangii* and related species. The BP tree is constructed, based on the combined matrix of ITS and *rps16* sequences. Numbers below branches are ML bootstraps and MP bootstraps and numbers above branches indicate Bayesian posterior probability. *Youngiahangii* is shown in bold.

**Figure 2.**
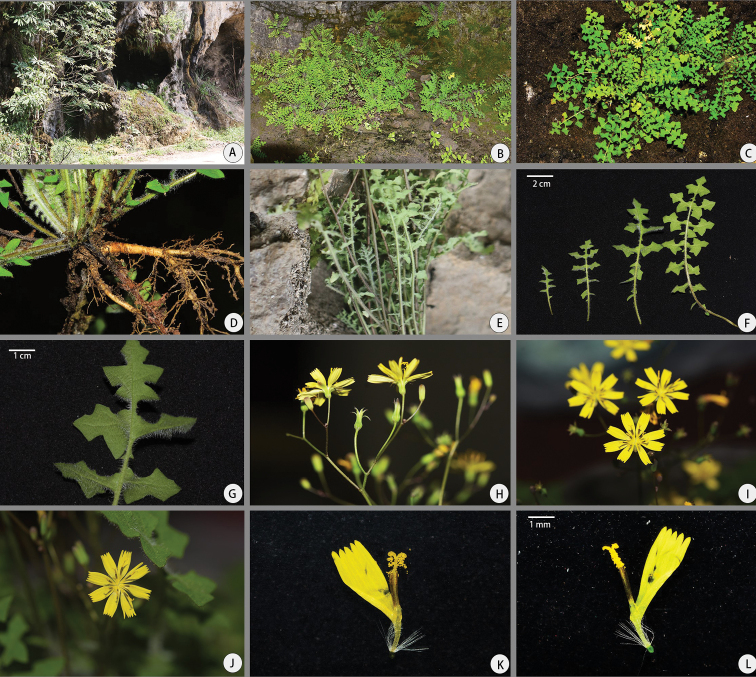
*Youngiahangii* T. Deng, D.G. Zhang, Qun Liu & Z.M. Li **A** habitat **B** population **C** habit **D** root **E** stems **F, G** leaves **H–J** capitula **K, L** floret (**A–E, H–J** Photos by D.F. Zhang **F, G, K, L** Photos by Qun Liu **D, E, J–M** HAC001 (KUN)).

##### Phenology.

Flowering and fruiting April to October.

##### Vernacular name.

五峰黄鹌菜, wǔ fēng huánɡ ān cài in Chinese Pinyin.

##### Etymology.

The species epithet honours Prof. Hang Sun (b. 1963), a Chinese botanist who has conducted research on plant taxonomy, floristics, biogeography and evolutionary biology and inspired many people through his work. He has also given a lot of support to the plant research work in Hubei.

##### Distribution and habitat.

*Youngiahangii* is known only from the type locality, Renheping in Wufeng Xian, Hubei, China; 500–1000 individuals are known along the edge of some small caves at the base of the karst hillside (Fig. 2A, B, C); at altitudes of 500–800 m.

##### Morphological assessment.

**Figure 3.**
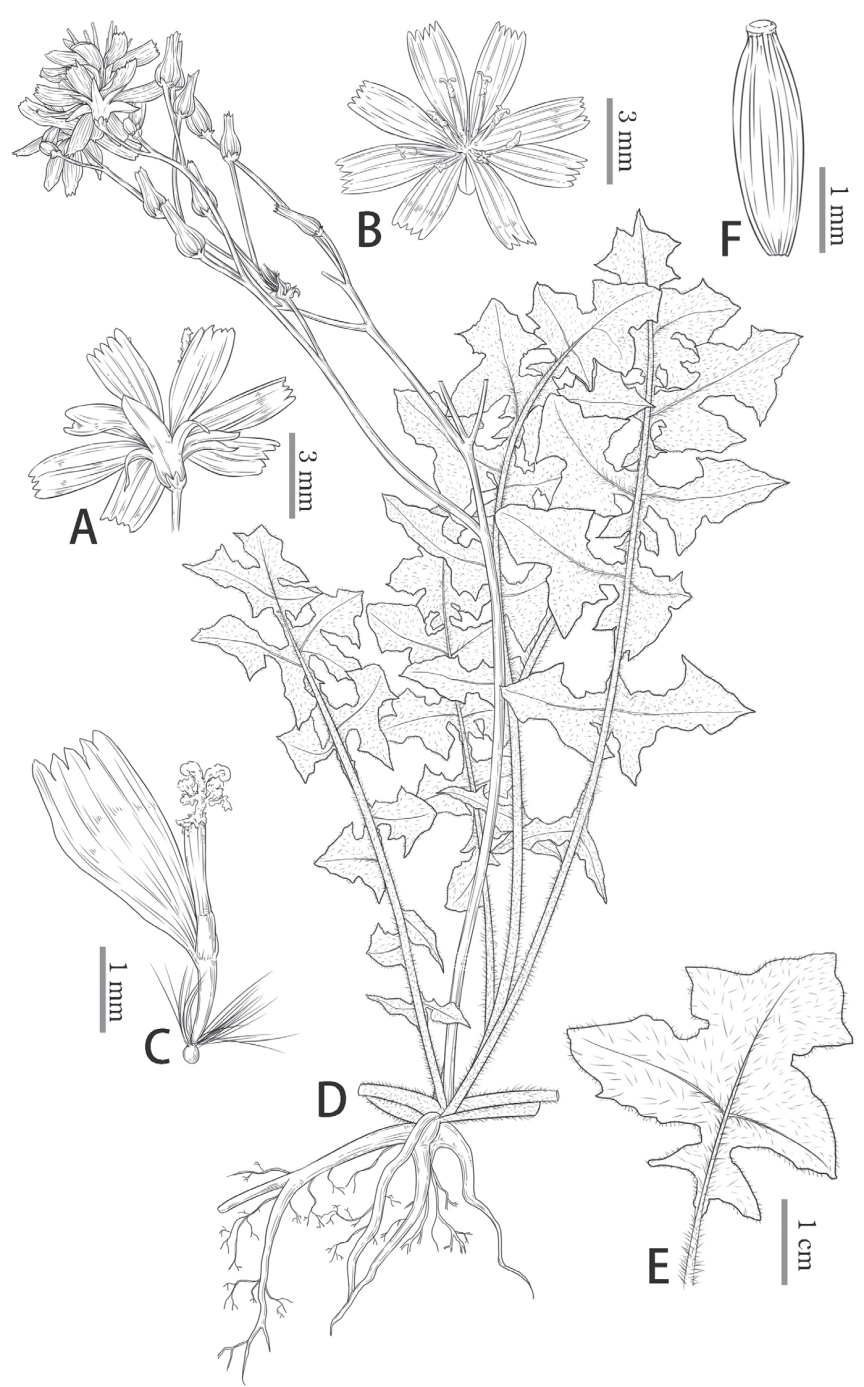
*Youngiahangii* T. Deng, D.G. Zhang, Qun Liu & Z.M. Li **A, B** capitula **C** floret **D** habit **E** leaf with hairs **F** achene (drawing by Jianing Yang).

Morphological characteristics suggest that *Y.hangii* is related to *Y.rubida* and *Y.heterophylla* owning 10–25 florets and resembles *Y.rosthornii* with bipinnately deeply partite leaves. The achenes of *Y.hangii* and *Y.rubida* are attenuated into a short beak, which is widened into the pappus disc. Several unique features including the shape, lobes and size of the leaves, the leaves with white simple hairs (Fig. 2E, F, G; Fig. 3F, G), phyllaries, number of florets and achenes differentiate *Y.hangii* from *Y.rubida* and *Y.heterophylla* (Table 1).

**Table 1.** Comparison of morphological characteristics between *Youngiahangii* and related species.

Character				* Y. hangii *	* Y. rubida *	* Y. heterophylla *	* Y. rosthornii *
Basal leaf	shape			oblanceolate, bipinnately partite	oblanceolate, pinnately deeply or completely partite	elliptic or oblong lanceolate, pinnately deeply or completely partite	long elliptic, bipinnately deeply partite with a large apical part
	lobes		shape	apical lobes halberd-shaped, apex acute, with a tapered tip, margin middle to deep lobed; lowest lobes narrowly triangular	apical lobes triangle, apex acute, with a tapered tip, margin serrate; lowest lobes serrate	apical lobes elliptic, irregularly elliptic, ovate or lanceolate, apex acute, with a tapered tip, margin entire, almost entire or serrate; lowest lobes narrowly halberd	apical lobes triangular, apex acute, with a tapered tip, margin entire, almost entire or serrate; lowest lobes narrowly triangular
			number of lateral lobes	5–10 pairs	2–3 pairs	1–8 pairs	5–7 pairs
	size			6–18 × 2–4 cm	3–7 × 1.5–3 cm	13–23 × 6–7 cm	20 × 8 cm
	surface			with white pubescent hairs on both surfaces, especially dense on veins	glabrous on both surfaces	sparsely pubescent on both surfaces	glabrous on both surfaces
Phyllaries				4 rows	4 rows	4 rows	4 rows
Number of florets				8–10	13–15	11–25	20
Achenes		colour		black	red	brown-purple	brown-purple
		shape		fusiform, attenuated into a narrow neck, with a conical beak	fusiform, attenuated into a narrow neck, with a conical beak	fusiform, attenuated into a narrow neck, without a beak	fusiform, attenuated into a narrow neck, without a beak
		length		2 mm	2.8 mm	3 mm	3.5 mm
		ribs		12–14 ribs with small bristles	12 ribs with small bristles	14–15 ribs with small bristles	14–15 ribs with small bristles
Pappus				white, rough, 3 mm	white, rough, 3.5 mm	white, rough, 3–4 mm	white, rough, 3.5 mm

##### Phylogenetic analysis.

The Bayesian tree showing PP support, ML bootstrap (LP) and MP bootstrap (BP) values for each clade are presented in Fig. 1. The species in clade Ι form a monophyletic group with PP = 0.97, but LP are with weak support and BP are in conflict with PP and they were instead with “–”, respectively in Fig. 1. *Youngiahangii* is nested within Clade I as sister to *Y.rubida* with strong support (PP = 1, LP = 83, BP = 88).

**Figure 4.**
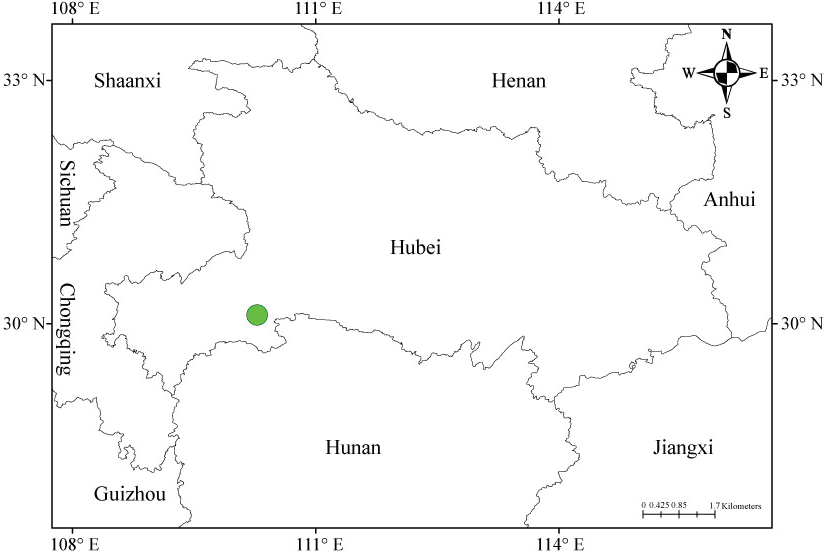
Distribution of *Youngiahangii* in Hubei Province, China.

## Discussion

Owning only 8–10 florets supports a placement of *Youngiahangii* in Y.sect.Youngia and its small involucres and achenes further support that *Y.hangii* is related to *Y.rubida*. However, there are some obvious differences between *Y.hangii* and *Y.rubida* and other species in the shape, lobes and size of the leaves and in white pubescent surfaces of the leaves. Moreover, *Y.rosthornii* also has bipinnately deeply partite leaves, but its leaves with a large apical part are different from *Y.hangii*.

Based on the combined datasets of the ITS and *rps16* sequences, BI, MP and ML trees with similar topologies were constructed. *Youngiahangii* was clustered with *Y.rubida* and nested in Y.sect.Youngia with strong support (PP = 1, LP = 87, BP = 74) and was sister to the clade of *Y.rubida* with strong support (PP = 1, LP = 83, BP = 88). The results from the phylogenetic analysis are consistent with the morphological comparisons. Although only one sample of *Y.hangii* was included in the phylogenetic analysis, *Y.hangii* and *Y.rubida* have obvious differences in morphology, so the morphological data and phylogenetic results altogether support our hypothesis of *Y.hangii* being a new species.

## Supplementary Material

XML Treatment for Youngia hangii

## Appendix I.

**Table A1.** Voucher information and GenBank accessions of species used in our study.

Species	Herbarium vouchers	GenBank accessions	
		*ITS*	*rps16*
* Askellia flexuosa *	*(1)*L.Peng & L.J.Tong1078(CDBI)*(2)*X.F.Gao,Z.M.Zhu,X.L.Zhao14954(CDBI)	KC968078 ^(1)^	KR733629 ^(2)^
*Crepidiastrumakagii* 1 = *Youngianansiensis* 1	*(1)*Y.L.Peng&L.J.Tong1151-1-3(CDBI)*(2)*Y. L.Peng & L.J.Tong1151*(3)*Y.L.Peng & L.J.Tong1131-3(CDBI)	KC968064 ^(1)^	KC968154 ^(2)^
*Crepidiastrumakagii* 2 = *Youngianansiensis* 2		KC968064 ^(1)^	KC968148 ^(3)^
* Crepidiastrum chelidoniifolium *	*(1)*~~	AB002627 ^(1)^	–
*Crepidiastrumdenticulatum* 1	*(1)*~*(2)*L.Pengpl2011052701	AB002623 ^(1)^	KC968107 ^(2)^
*Crepidiastrumdenticulatum* 2		AB002622 ^(1)^	KC968107 ^(2)^
*Crepidiastrumdiversifolium* 1	*(1)*L.Pengpl2011072605-6(CDBI)*(2)*L.Pengpl2011072804*(3)*L.Pengpl2011072605	KC968072 ^(1)^	KC968150 ^(2)^
*Crepidiastrumdiversifolium* 2		KC968072 ^(1)^	KC968149 ^(3)^
* Crepidiastrum lanceolatum *	~	AB002624	AB598601
* Crepidiastrum pinnatipartitum *	*(1)*Y.L.Peng325-6*(2)*Y.L.Peng325	KC968061 ^(1)^	KC968151 ^(2)^
* Crepidiastrum platyphyllum *	*(1)*K2-CR 1267*(2)*~	AY876264 ^(1)^	AB598599 ^(2)^
*Crepidiastrumsonchifolium* 1	*(1)*SCSB-JS0086*(2)*Lilan245	MH808121 ^(1)^	–
*Crepidiastrumsonchifolium* 2		MH808120 ^(2)^	–
*Crepidiastrumtaiwanianum* 1	*(1)*~	AB002615 ^(1)^	–
*Crepidiastrumtaiwanianum* 2		AB002614 ^(1)^	–
* Crepidiastrum tenuifolium *	*(1)*~	EU363645 ^(1)^	–
* Scorzonera austriaca *	*(1)*Y.L.Peng & L.J.Tong1123-3-1(CDBI)*(2)*Y.L.Peng & L.J.Tong1123-2(CDBI)	KC968059 ^(1)^	KC968135 ^(2)^
*Youngiacineripappa* 1	*(1)*J.W. Zhang & W.D. Zhu ZZ09041 (KUN)*(2)*Z.M.Zhu682(CDBI)*(3)*~	LT722046 ^(1)^	KR733617 ^(2)^
*Youngiacineripappa* 2		LT722046 ^(1)^	KF732162 ^(3)^
*Youngiaerythrocarpa* 1	~	AB598566	KF732132
*Youngiaerythrocarpa* 2		AB598566	KF732169
*Youngiagracilipes* 1	*(1)*X.F.Gao14517-9*(2)*L.Pengpl2011082607-2*(3)*X.F.Gao14517	KC968076 ^(1)^	KC968155 ^(2)^
*Youngiagracilipes* 2		KC968076 ^(1)^	KC968126 ^(3)^
*Youngiaheterophylla* 1	*(1)*~*(2)*X.F.Gao,Y.L.Peng,B.Xu & X.Zheng11937-2*(3)*X.F.Gao,Y.L.Peng,B.Xu & X.Zheng11694	AB598561 ^(1)^	KC968123 ^(2)^
*Youngiaheterophylla* 2		AB598561 ^(1)^	KC968122 ^(3)^
*Youngiahumifusa* 1	*(1)*Y.L.Peng & L.J.Tong981-3-1*(2)*Y.L.Peng & L.J.Tong1012*(3)*X.F.Gao, Y.L.Peng, B.Xu & X.Zheng13147(CDBI)*(4)*Y.L.Peng & L.J.Tong981-1	KC968034 ^(1)^	KC968115 ^(3)^
*Youngiahumifusa* 2		KC968035 ^(2)^	KC968113 ^(4)^
Youngia japonica subsp. formosana	*(1)*~	AB598559 ^(1)^	–
Youngiajaponicasubsp.japonica 1	*(1)*~*(2)*ZhangJW 388 (KUN)*(3)*L.Pengpl2011072001-6*(4)*X.F.Gao,Y.L.Peng,B.Xu & X.Zheng11605	AB598557 ^(1)^	KC968153 ^(3)^
Youngiajaponicasubsp.japonica 2		HQ436229 ^(2)^	KC968118 ^(4)^
* Youngia longiflora *	*(1)*~	AB598558	–
*Youngiapaleacea* 1	*(1)*~*(2)*~*(3)*L.Peng pl2011082701(CDBI)*(4)*L.Peng pl082605-1(CDBI)	KJ502310 ^(1)^	KR733620 ^(3)^
*Youngiapaleacea* 2		KJ502311 ^(2)^	KR733616 ^(4)^
* Youngia rubida *	*(1)*Y.L.Peng93-3(CDBI)*(2)*Y.L.Peng pl081201(CDBI)	KC968048 ^(1)^	KR733627 ^(2)^
* Youngia hangii *	*(1)*HHE 3256(KUN)	MZ817057	MZ923644
* Youngia simulatrix *	*(1)*~	KJ502312 ^(1)^	–
* Youngia szechuanica *	*(1)*~	KJ502313 ^(1)^	–
* Youngia thunbergiana *	*(1)*~	KC539465 ^(1)^	–
* Youngia zhengyiana *	*(1)*~	KJ502314 ^(1)^	–
